# Ultrastructural and Molecular Changes in the Developing Small Intestine of the Toad *Bufo regularis*

**DOI:** 10.1155/2014/986784

**Published:** 2014-03-13

**Authors:** S. A. Sakr, G. M. Badawy, H. T. El-Borm

**Affiliations:** Department of Zoology, Faculty of Science, Menoufiya University, Shebeen El-Koom, Egypt

## Abstract

The ontogenetic development of the small intestine of the toad *Bufo regularis* was investigated using twofold approaches, namely, ultrastructural and molecular. The former has been done using transmission electron microscope and utilizing the developmental stages 42, 50, 55, 60, 63, and 66. The most prominent ultrastructural changes were recorded at stage 60 and were more evident at stage 63. These included the appearance of apoptotic bodies/nuclei within the larval epithelium, the presence of macrophages, swollen mitochondria, distorted rough endoplasmic reticulum, chromatin condensation, and irregular nuclear envelop, and the presence of large vacuoles and lysosomes. The molecular investigation involved examining DNA content and fragmentation. The results showed that the DNA content decreased significantly during the metamorphic stages 60 and 63 compared with both larval (50 and 55) and postmetamorphic (66) stages. The metamorphic stages (60 and 63) displayed extensive DNA laddering compared with stages 50, 55, and 66. The percentage of DNA damage was 0.00%, 12.91%, 57.26%, 45.48%, and 4.43% for the developmental stages 50, 55, 60, 63, and 66, respectively. In conclusion, the recorded remodeling of the small intestine represents a model for clarifying the mechanism whereby cell death and proliferation are controlled.

## 1. Introduction

During amphibian metamorphosis, the body of the tadpole undergoes remodeling from the larval to the adult form suitable for terrestrial life. In amphibians, as in many other taxa, intestinal length is often an indicator of diet [1]. Therefore there is a general relationship between the length of the intestine and feeding habits. During development, herbivorous feeding of the anuran larva is superseded by carnivorous feeding in the postmetamorphic froglet and adult [2]. In the amphibian small intestine, the epithelial transformation from the larval to adult type is mainly the result of degeneration of the larval epithelium and development of the new (adult) epithelium. The mechanism that regulates the balance between degeneration of the primary larval epithelium and development of the secondary adult epithelium in the metamorphosing small intestine is very interesting with regard to developmental biology but remains largely unknown [3]. Cell death is one of the most essential components of amphibian remodeling and has been thought of as being “programmed” in the genetic sense [4]. It occurs in a variety of organs during amphibian metamorphosis and is usually identified by electron microscopy as apoptosis [5–7]. Cell death observed during amphibian metamorphosis shares common characteristics of apoptosis as follows: condensation and margination of chromatin against the nuclear envelope followed by formation of membrane-bounded cell globules, that is, apoptotic bodies which contain intact cell membranes and organelles, and a fragmented nucleus.

Although there have been numerous studies published on the degeneration of amphibian tadpoles, they have often confused the death process at the cellular level with that occurring at the tissue or organ level. The majority of cell death, reports have been mainly focused on histolytic changes such as development of lysosomes [8]. A number of methods were recently developed for visualizing individual cells undergoing programmed cell death that is, apoptosis on histological sections, and consequently it is now easy to distinguish cells undergoing apoptosis in situ by both light and electron microscopy [9]. Apoptosis is an active form of cell death characterized by biochemical and morphological processes, especially by chromatin condensation, polynucleosomal DNA fragmentation, and the fragmentation of the cell into apoptotic bodies [4]. Using transmission electron microscopy Ishizuya-Oka [5] has indicated that apoptosis is involved in the small intestinal remodeling in* Xenopus*. However, the distribution pattern of apoptosis in the small intestine during metamorphosis has not been precisely examined so far, because morphological changes of apoptosis can be detected by electron microscopy for only a short period of time [4, 10].

Degenerative cellular changes with the developing small intestine occur during a short period around the onset of metamorphic climax. For example, the microvilli composing the brush border decrease in number and height, whereas lysosomes increase in number and in hydrolytic activity [11]. Just after the beginning of the primary epithelial degeneration, adult epithelial primordia are detected at the epithelial-connective tissue interface as small islets consisting of undifferentiated epithelial cells [12]. The primordia rapidly grow and invaginate into the surrounding connective tissue through active cell proliferation and differentiate to form the secondary adult epithelium, replacing the degenerating primary larval epithelium [13]. As morphogenesis of the intestinal folds proceeds, adult epithelial cells differentiate into major absorptive epithelial cells, goblet cells, and endocrine cells, that is, into all the cell types of mammalian intestinal epithelium except Paneth cells [14].

Many investigators are interested in rapid assays to detect and quantify apoptosis in cell populations. It is possible to quantify DNA fragmentation during apoptosis [15]. Furthermore, flow cytometry can be used to detect several of the morphological changes characteristic of cells undergoing apoptosis. It can be used to follow the percentage of apoptotic cells present in a cell population over the course of an experiment [16].

Phases in the Egyptian toad ontogeny have been mostly established according to the external features [17]. However, it is well known that the embryonic, larval, and different metamorphic periods of anurans involve extensive developmental changes in the internal organs [6, 18]. Among the latter, the small intestine lacks a detailed investigation in terms of both ultrastructural and molecular changes. This stimulated us to study the possible ultrastructural and molecular changes of the developing small intestine before, during, and after spontaneous metamorphosis.

## 2. Materials and Methods

### 2.1. Animals and Care

Principles of animal care as well as all experimental aspects of this work were conducted in compliance with the institutional guidelines for the care and use of animals. Several ribbons of fertilized eggs of the toad,* Bufo regularis,* were brought into the laboratory from the fields of Shebeen El-Koom districts during the breeding season which lasts from March to September. Developing eggs were collected in a mesh-collecting basket and shipped in plastic bags filled with dechlorinated tap water. The ribbons were divided into small bunches and kept in either white enamel-coated pans of 30 × 20 × 3.5 cm., provided with two liters of dechlorinated tap water, or glass aquaria with sufficient supply of dechlorinated tap water so that the water depth remained equal in both cases. On reaching the feeding stage 44, the tadpoles were redistributed in a smaller number per rearing container, using additional supply of pans. From the initiation of feeding until the end of the aquatic period of development, throughout the study, tadpoles were fed* ad libitum* either freshly or frozen boiled spinach until the beginning of the metamorphic climax phase (stage 59) at which the animals stopped feeding until reaching stage 66. Rearing water was changed every necessitation that was at least once weekly. Starting from stage 56, tadpoles were transferred into other pans with shallower level of water and small pieces of stones. When the second forelimb emerged (stage 59) and following changing-over to air breathing, tadpoles were removed from the pans and housed individually in a labeled bowl with perforated cover and provided with a piece of moistened cotton on the floor. Development was monitored until the tail bud had completely, absorbed, that is, reaching stage 66. Rearing took place at room temperature that was 28 ± 2°C.

### 2.2. Sampling

A number of developmental stages were selected based on our previous study [18] conducted on the same experimental model. For staging purposes, the normal table of Sedra and Michael [17] which is specific for* Bufo regularis* was utilized as a morphological guide. Starting from stage 42 and after discarding any abnormal individuals, specimens were collected randomly from each tank, had a prolonged anesthetization in 250 mg/L MS222, (tricaine methane sulphonate, Sigma, St. Louis, Mo, USA) and staged. The small intestine of the concerned developmental stages was dissected and treated differently according to the type of investigation. The study was based mainly on the developmental stages 42 (3 days 6 hour), 50 (17 days), 55 (26 days), 60 (41 days), 63 (43 days), and 66 (46 days). However, it was infeasible to include stage 42 when conducting the molecular investigation. A total of 130 animals were used throughout the present investigation (30 for ultrastructural investigation; 30 for DNA content; 35 for DNA fragmentation and 35 for flow cytometry). Taken our previous investigation [18] as a guide, it was found that the percentage of shortening of the anterior part and the posterior part of the small intestine exhibits a slight variation, so the anterior part of the small intestine was chosen for ultrastructure investigation, while the molecular investigation was conducted on the whole small intestine.

#### 2.2.1. Ultrastructural Investigation

For transmission electron microscopical investigation, the method of Glauert [19] was followed. Briefly, samples of 2 mm pieces of the anterior part of the small intestine just posterior to the entrance of the hepatopancreatic duct were excised in different developmental stages, that is, 42, 50, 55, 60, 63, and 66. The pieces were fixed rapidly for 4 hours at room temperature in Karnovsky fixative [20].

After rinsing in phosphate buffer, samples were postfixed in buffered solution of 1% osmium tetraoxide for three hours at 4°C. The specimens were then washed in phosphate buffer several times for 10 minutes. This was followed by dehydration in ascending grades of ethanol and transferring to a solution of propylene oxide for clearing. The samples were then infiltrated in a mixture of propylene oxide and epon (1 : 1). After infiltration, tissues were embedded in the epoxy resins using beam capsules and blocks were prepared. Semithin sections of 1 *μ*m thickness stained with Toluidine blue were produced for light microscopical examination. After determining the desired areas from the semithin sections, ultrathin (50 nm) sections were cut, mounted on formvar-coated grids, and stained with uranyl acetate for 10 minutes. Sections were then stained with lead citrate for 10 minutes. Examination of grids was done by using JEOL electron microscope, Electron Microscope Unite, Tanta University, Tanta, Egypt. Selected sites were digitally photographed and then printed on Kodak sensitive printing paper.

#### 2.2.2. Molecular Investigation

*Determination of DNA Content*. Tissue DNA content was measured by the method of Burton [21]. Briefly, tissue was homogenized (10% in normal saline), then 0.5 mL of 0.5 N perchloric acid. The mixture was kept in water bath, maintained at 90°C for 15 minutes with gentle shacking to facilitate the quantitative separation of nucleic acids from proteins and degrade the nucleic acids mainly to soluble nucleotides. The tubes were then centrifuged for 3 minutes at 3000 rpm. The obtained supernatant was used for the estimation of total DNA. An amount of 1 mL diphenylamine was added to the supernatant. The tube was kept in a boiling water bath for 20 minutes. Thereafter, the tubes were kept in an ice bath until reaching room temperature. By that time, the blue color was developed and read against blank (tube contain 0.5 mL perchloric acid + 1 mL diphenylamine) at 640 nm. Since there is a linear relationship between absorbance and DNA concentration, the total DNA content was extracted using a standard curve, where absorbance values at 600 nm that constitute 0.1, 0.2, 0.3, 0.4, and 0.5 are equivalent to 50, 150, 250, 350, 450, and 550 *μ*g/mL DNA concentration, respectively.

*Determination of DNA Fragmentation*. As a measure of apoptotic DNA fragmentation, the presence of DNA ladder was determined according to Wlodek et al. [22]. Extraction of DNA was done according to the method of Aljanabi and Martinez [23]. Briefly, biopsies of the small intestine weighing 10 mg were squeezed in Eppendorf tubes, lysed with 600 microlitre (*μ*L) buffer (50 mM NaCl, 1 mM Na_2_EDTA, and 0.5% sodium dodecyl sulphate, pH 8.3), and shacked gently. The mixture was incubated overnight at 37°C. For protein precipitation, an amount of 200 *μ*L of saturated NaCl was added to the samples, shacked gently, and centrifuged at 12000 rpm for 10 minutes. The supernatant was transferred to new Eppendorf tube and the DNA was precipitated by 600 *μ*L cold isopropanol. The mixture was inverted several times till fine fibers of nucleic acids appeared, at which time the mixture wss centrifuged for 5 minutes at 12000 rpm. The supernatant was then removed and the pellets (DNA and RNA) were washed with 500 *μ*L 70% ethanol and centrifuged at 12000 rpm for 5 minutes. The supernatant was decanted or tipped out and the tubes were plotted on Whatman paper to dry for 10 minutes. The pellets were resuspended in 50 *μ*L of TE buffer (10 Mm Tris, 1 mM EDTA, pH 8). The resuspended DNA was incubated for 30–60 minutes with loading mix (Rnase + loading buffer) and then added into the agarose gel wells. A gel was prepared with 2% electrophoretic grade agarose containing 0.1% ethidium bromide (200 *μ*g/mL). The DNA samples were mixed with loading buffer (0.25% bromophenol blue, 0.25% xylene cyanol Ff, and 30% glycerol) and loaded into the wells (2 *μ*g of DNA/Lane) with a standard molecular-sized ladder marker (Pharmacia Biotech, USA). The gel was electrophoresed at a current of mA for 2.5 hours using the submarines gel electrophoresis machine. The DNA was visualized and photographed with illumination under ultraviolet (UV) light using a photodocumentation hood (Fisher Scientific, Pittsburgh, PA, USA) equipped with a polaroid 667 film with an orange filter (Kodak, Rochester, NY, USA). The UV reacts with the ethidium bromide to show the DNA fragments. Apoptotic bands appeared and located at 200 bp and its multiples.

*Flow Cytometry*. To analyze the degree of apoptosis within the selected developmental stages, flow cytometry was employed. Detection of DNA damage and measurement of cell cycle analysis by flow cytometry have been conducted in the whole small intestine. Samples were transported to the laboratory and prepared according to Tribukait et al. [24]. Briefly, tissue was homogenated and suspended in phosphate buffer saline (PBS) and the cell suspension was centrifuged at 2000 rpm for 10 min. The supernatant was decanted. Flow cytometry (Becton Dickinson, Sunnyvale, CA, USA) analysis was performed on single cell suspensions washed three times with PBS. After washing, the cell viability was determined and apoptosis was measured by using the sub-G1 peak staining with propidium iodide [25]. The average number of evaluated nuclei per specimen was 20000 and the number of nuclei scanned was 120 per second. DNA histogram derived from flow-cytometry was obtained with a computer program for Dean and Jett mathematical analysis [26]. Data analysis was conducted using DNA analysis program MODFIT (Verity Software House, Inc., Topsham, ME, USA, version 2.0, power Mac with 131072 KB).

### 2.3. Statistical Analysis

All data sets were expressed as mean ± standard error of the mean (SEM). The data were analyzed statistically for normal distribution (student'-*t*-test) and homogeneity of variances (Levene' test) using SPSS software for Windows, version 11. The significances of the obtained data were classified into three categories according to *P* values; that is, *P* < 0.0001, *P* < 0.03, and *P* < 0.05.

## 3. Results

### 3.1. Ultrastructural Observations

**Figure 1:**
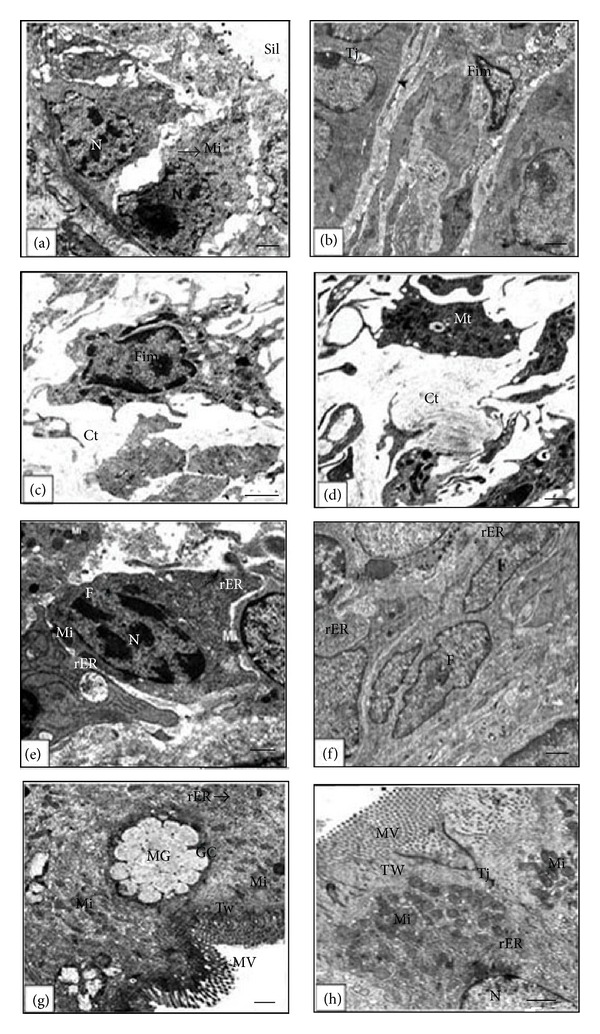
Electron micrographs of the anterior portion of the small intestine of the larval stage 42 ((a)–(d)) and the premetamorphic stage 50 ((e)–(h)) showing the following. (a) and (b): epithelial cells contact to the basal lamina (arrow head). (c): an immature fibroblast-like cell (Fim) with large nuclei and poorly developed cell organelles. (d): a mitotic cell (Mt) in the connective tissue. (e): a fibroblast with well-developed rough endoplasmic reticulum (rER). (f): connective tissue (Ct) contains a fibroblast (F) with well-developed rER. (g): a goblet cell (GC) containing mucous granules (MG) in the apical surface of the larval epithelium. (h): supranuclear cytoplasm of two adjacent columnar cells with uniform microvilli (MV), numerous mitochondria (Mi), and vesiculated rough endoplasmic reticulum (rER) beneath a well-developed terminal web (TW). The lateral junctional complex joining neighboring cells is visible. Scale bar = 1 *μ*m.

The ultrastructural results showed that the general architecture of the small intestine is correlated with the state of development, that is, larval, metamorphic, and postmetamorphic. It therefore displayed different characteristic features depending on the investigated developmental stage starting from the larval stage 42 and ending with the postmetamorphic stage 66. Because both the epithelium and the connective tissue of the prefeeding stage 42 are unfunctional, they were poorly developed. The simple columnar epithelial cells had large basal nuclei and poorly developed microvilli (Figure 1(a)) and were separated from the connective tissue by a thin continuous basal lamina (Figure 1(b)). The cell density of connective tissue was low in every region (Figures 1(c) and 1(d)). Almost all of the cells observed by electron microscopy within the connective tissue were immature fibroblast-like cells possessing large nuclei, irregular shape, and poorlydeveloped cell organelles. Also within the connective tissue, there were few randomly distributed mitotic cells (Figure 1(d)).

At stage 50 and as the small intestine switched to be functional, the columnar absorptive epithelial cells were evident. The connective tissue had specific fibroblasts characterized by well-developed rough endoplasmic reticulum and mitochondria (Figures 1(e) and 1(f)). The most prominent feature was the columnar absorbing epithelium with a well-developed microvilli compared with the previous stage. The microvilli appeared as a series of finger like projections which extend into the lumen (Figure 1(g)). Goblet cells were readily distinguishable from other types of epithelial cell by their mucous granules, which often fill the cytoplasm between the basally located nucleus and their luminal surface which was devoid of microvilli (Figure 1(g)). The underlying cytoplasmic zone which continued with the microvilli, that is, the terminal web, contained fibrillar material and was devoid of organelles. Beneath the terminal web, numerous mitochondria, electron-dense granules, small smooth vesicles, and rough endoplasmic reticulum were fairly distributed within the cytoplasm (Figure 1(h)).

**Figure 2:**
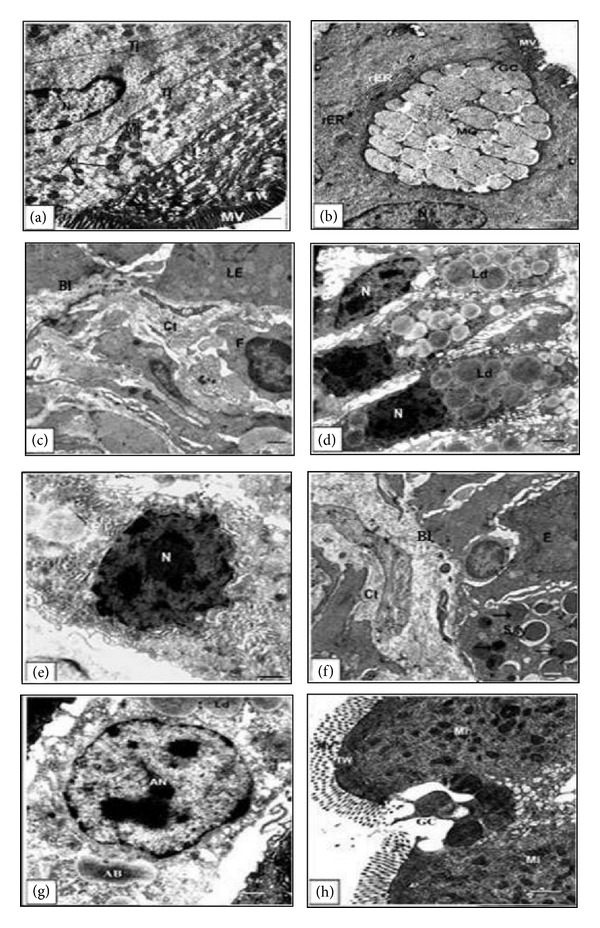
Electron micrographs of the anterior portion of the small intestine of the prometamorphic stage 55 ((a)–(c)) and the metamorphic stage 60 ((d)–(h)) showing the following (a): apical cytoplasm with a well-developed microvilli (MV), numerous mitochondria (Mi), terminal web (TW) and tight junction (Tj). (b): the apical surface of the epithelium containing large goblet cell (GC) with mucus granules (MG). (c): epithelial-connective tissue interface showing the thin basal lamina (BL) under the larval epithelial (LE) cells. (d): a columnar epithelium with numerous lipid droplets (Ld). (e): intestinal epithelial cell with irregular nuclear envelop and chromatin condensation. (f): epithelial-connective tissue interface showing apoptotic bodies (arrows) in the larval epithelium. (g): apoptotic nucleus (AN) with chromatin condensation and irregular nuclear envelop. (h): discharging GC at the apical region. Scale bar = 1 *μ*m.

Throughout the prometamorphic stage 55, no adult epithelial primordia were detected and the epithelium remained morphologically defined as the larval type. As seen in Figures 2(a) and 2(b), the larval epithelium still differentiated into columnar absorptive cells which retained well-developed brush border and goblet cells. The apical cytoplasm of the columnar absorbing cells is crowded with mitochondria (Figure 2(a)). The basal lamina of this stage was relatively thicker than that of the other previous stages, that is, the larval stages 42 and 50 (Figure 2(c)). As far as the ultrastructural investigation can tell, there were no apoptotic cells.

At stage 60, the nuclei were large in relation to the amount of surrounding cytoplasm which had numerous lipid droplets (Figure 2(d)). General examination of the fine structure of the apical cytoplasm brings to light details of their structure in which they exhibit differences from functional larval cells. Therefore, most larval epithelial cells had nuclei characterized by irregular nuclear envelop and chromatin condensation. The cell organelles including rough endoplasmic reticulum and mitochondria were generally distorted (Figure 2(e)) and the microvilli were shorter; in some instances they are fewer and are not always perpendicular to the cell surface. A large number of the larval epithelial cells displayed variable signs of apoptosis and consequently apoptotic bodies of variable sizes can be seen (Figure 2(f)). The larval epithelium at this stage was characterized by numerous intraepithelium macrophages filled with many inclusions. This metamorphic climax stage was ultrastructurally characterized by a thickening of the basal lamina caused by vigorous folding in every region just beneath both types of epithelia (Figure 2(f)). Some of the apoptotic cells were located in the intestinal lumen and characterized by chromatin condensed nuclei and irregular nuclear envelop (Figure 2(g)). Goblet cells are numerously scattered throughout the gut epithelium and their discharging activity was prominent (Figure 2(h)).

**Figure 3:**
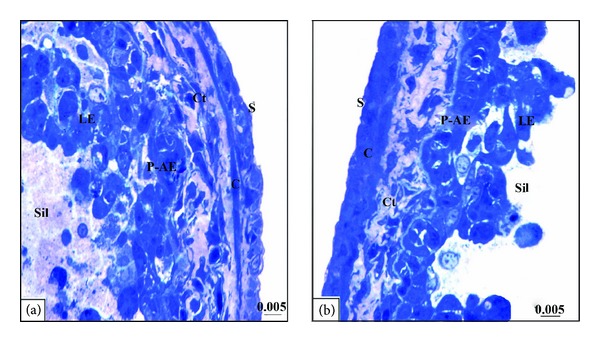
((a)-(b)) Photomicrographs of transverse semithin sections of stage 63 stained with Toluidine blue showing the apoptotic larval epithelium cells and cellular debris within the small intestinal lumen. S: serosa; C: circular muscle layer; Ct: connective tissue; P-AE: adult epithelial primordia; LE: larval epithelium; Sil: small intestinal lumen. The length unit for scale bars is millimeter.

**Figure 4:**
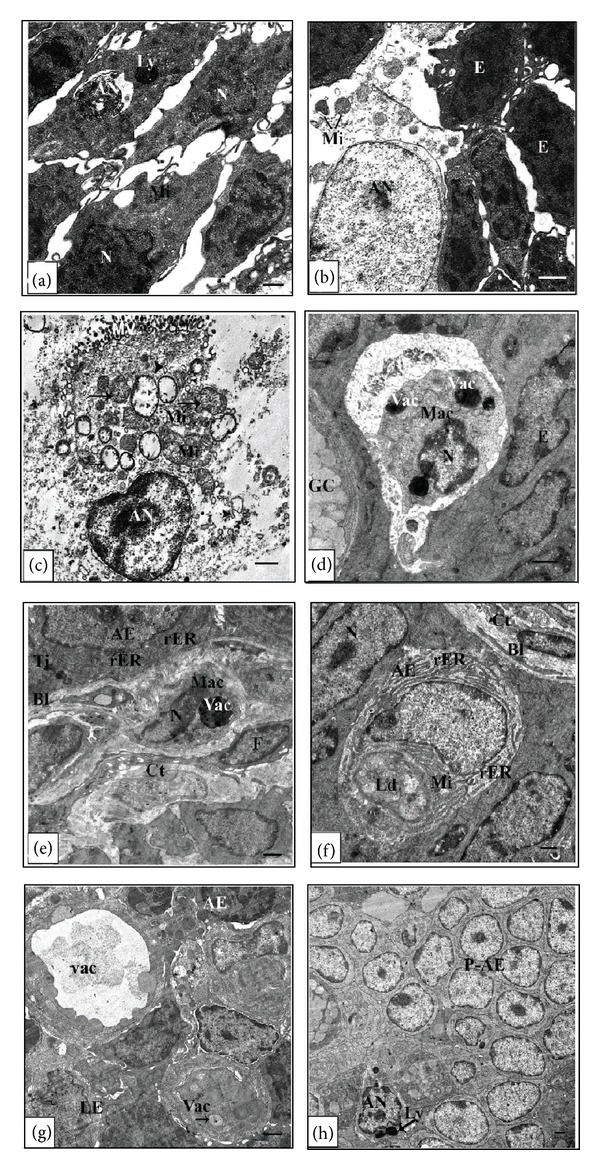
Electron micrographs of the anterior portion of the small intestine of the metamorphic stage 63 ((a)–(h)) showing the following (a): apoptotic nucleus (AN) and lysosomes. (b): degenerating cell with AN. Although the plasma membrane is disrupted, the mitochondria (Mi) retain normal morphological structure. (c): degenerating epithelial cell with AN characterized by chromatin condensation and irregular nuclear envelop. The rough endoplasmic reticulum (rER) was evidently degenerated (arrow heads) and the Mi were swollen (arrows). (d): an intraepithelial macrophage (Mac) with numerous large vacuoles (Vac) and pseudopodium-like processes. (e): phagocytic Mac possessing large Vac in the connective tissue (Ct). F: adult epithelial-connective tissue interface. Epithelial cell contains Mi and numerous rER. The basal lamina (BL) is thick. (g): coexistence of larval (LE) and adult epithelia (AE) at stage 63. Vac are localized in the LE. (h): adult epithelial primordia (P-AE) replace the LE, where AN are detected at stage 63. Scale bar = 1 *μ*m.

At metamorphic stage 63 cellular debris were easily detected in the intestinal lumen of the semithin sections (Figures 3(a) and 3(b)). At the ultrastructural level, the larval epithelium exhibited dramatic changes as the apoptotic nuclei were frequently seen (Figures 4(a) and 4(b)). The functionless small intestine of this stage displayed several features of degeneration. Some degenerating cells had apoptotic nucleus with disrupted plasma membrane, distorted rough endoplasmic reticulum and mitochondria. However, there were apoptotic cells which retained some normal organelles (Figure 4(b)), while others had apoptotic nucleus with chromatin condensation and irregular nuclear envelop in addition to degenerated rough endoplasmic reticulum, and swollen mitochondria (Figure 4(c)). Intraepithelial macrophages containing large vacuoles and having pseudopodium-like processes were frequently observed (Figure 4(d)). The number of macrophages increased concomitantly with larval cell death. Within the connective tissue there were phagocytic macrophages with large vacuoles (Figure 4(e)). Through the examination, it was noticed that there is a variation in the sizes of macrophages which were small in the connective tissue but large within the epithelium. The adult epithelial cells with mitochondria and rough endoplasmic reticulum were resting on a thick basal lamina (Figure 4(f)) and the connective tissue showed a rapid increase in cell number.

The coexistence of both larval and adult epithelia was evident at stage 63 (Figure 4(g)). Concomitantly with the sudden increase in the number of apoptotic cells in the larval epithelium, active cell proliferation started in the adult epithelial primordia. This active proliferation of the adult epithelial primordia leaded to the formation of nests of adult epithelial cells to replace the degenerative larval epithelium (Figure 4(h)). These nests clearly have their origin within the epithelium since they are separated from the underlying connective tissue by a basal lamina. Closer examination of the ultrastructure revealed that apoptosis of the larval epithelium and active cell proliferation of the adult epithelium coincide with a temporary vigorous folding of the basal lamina underlying the epithelia. This indicates that both these processes are regulated by the modification of the basement membrane components.

**Figure 5:**
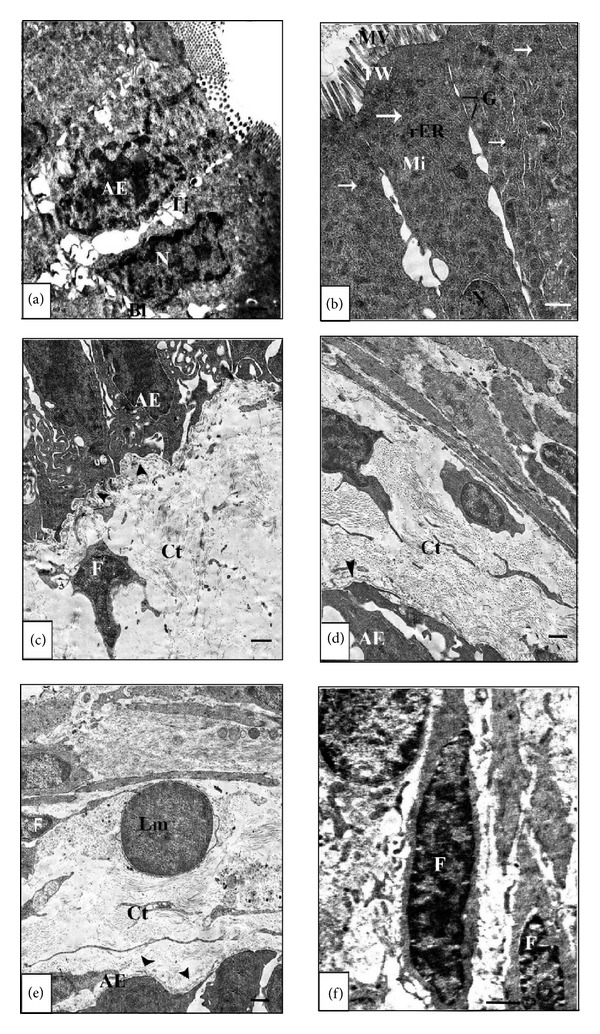
Electron micrographs of the anterior portion of the small intestine of the postmetamorphic stage 66 showing the following. (a): adult intestinal epithelial (AE) cells rest on a thin basal lamina (BL). (b): columnar cells of microvilli (MV) project into the intestinal lumen. A terminal web (TW) can also be seen. The lateral cell borders of two adjacent cells are joined near their apices by a series of desmosomes (D). Mitochondria (Mi) (arrows) and rough endoplasmic reticulum (rER) are present beneath the specialized cell border. (c) and (d): adult epithelium and connective tissue interface. The BL (arrow head) is thin and continuous. (e): adult epithelium and connective tissue interface. The lymphocyte (Lm) in the connective tissue (Ct). (f): an ordinary fibroblast (F) surrounded by collagen fibers in the Ct. Scale bar = 1 *μ*m.

At the postmetamorphic stage 66, the adult epithelial cells were arranged in a single layer and contained medium to basally located nuclei. There was no trace for the apoptotic larval epithelial cells. The adult columnar epithelial cells were elongated resting on a thin basal lamina (Figure 5(a)) and their apical part had regular and long microvilli which projected into the intestinal lumen forming the brush border (Figure 5(a)). The terminal web was rich in mitochondria and rough endoplasmic reticulum (Figure 5(b)). The basal lamina of this stage was very thin compared to the previous metamorphic stages. Figures 5(c) and 5(d) demonstrate the thin basal lamina and the well differentiated epithelial cells and connective tissue. A few lymphocytes were detected within the connective tissue (Figure 5(e)) which possessed well differentiated spindle fibroblasts (Figure 5(f)).

### 3.2. Total DNA Content

**Figure 6:**
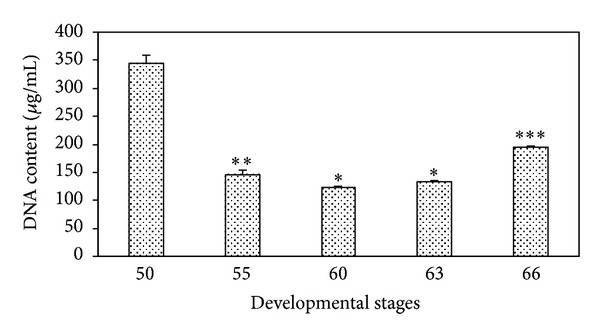
Changes in the DNA content before, during, and after metamorphosis in the developing small intestine of the toad* Bufo regularis*. Data are represented as mean ± SEM (*n* = 6).  ***P < 0.0001, **P < 0.001, and  *P < 0.05.

Figure 6 shows the mean values of the DNA content before, during, and after metamorphosis in the developing small intestine. The DNA content exhibited evident high values at the premetamorphic stage 50 (343.83 ± 14.93 *μ*g/mL) compared with the other more advanced developmental stages. Therefore, there was an evident decline in the DNA content at the prometamorphic stage 55 (146.50 ± 7.91 *μ*g/mL) and the metamorphic stages 60 and 63 (123.33 ± 1.63 *μ*g/mL and 132.33 ± 2.99 *μ*g/mL, resp.) followed by an increase in the postmetamorphic stage 66 (194.91 ± 1.47 *μ*g/mL).

### 3.3. DNA Fragmentation Pattern

**Figure 7:**
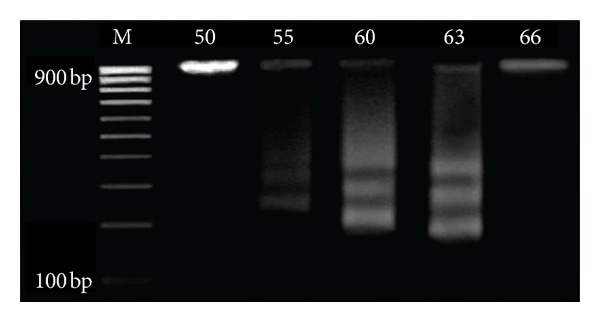
Agarose gels showing evident variations of the DNA fragmentation in extracts from the whole small intestine of stages 50 (Lane 2), 55 (Lane 3), 60 (Lane 4), 63 (Lane 5), and 66 (Lane 6), M (marker).

As can be seen in Figure 7, the small intestine of the premetamorphic stage 50 exhibited no sign of DNA fragmentation. Consequently, the first signs of DNA fragmentation were observed at the premetamorphic stage 55. In the metamorphic stages 60 and 63, DNA degradation was more extensive and the banding pattern was clearer with evident broad peaks. Therefore, the metamorphic stages displayed extensive DNA laddering compared with the developmental stages 50 and 66 where the DNA appeared to be intact with no sign for fragmentation. The lanes which represent the metamorphic stages 60 and 63 displayed evident ladders of DNA fragments. The postmetamorphic stage 66, on the other hand, displayed obvious similarity with the premetamorphic stage 50 in terms of the evident lack of DNA fragmentation.

### 3.4. Flow Cytometry

**Figure 8:**
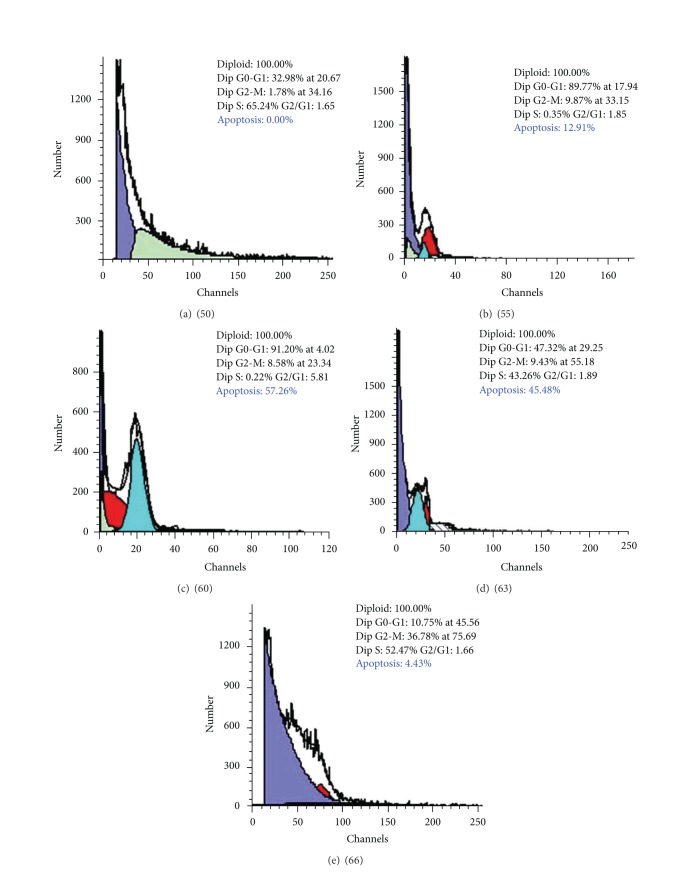
Computerized cell cycle analysis (flow cytometry) for apoptosis in the small intestinal cells during different developmental stages (50, 55, 60, 63, and 66, resp.) of the toad* Bufo regularis*.

**Figure 9:**
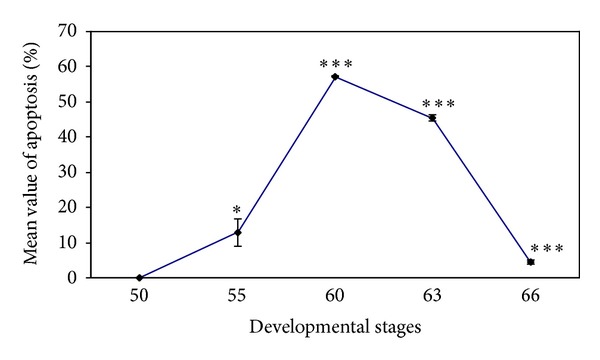
Graph showing the correlation between the percentage of DNA damage and the investigated developmental stages within the small intestine using cell cycle analysis. Data are represented as mean ± SEM (*n* = 6). ***P < 0.0001,  *P < 0.05.

The outcome of the cell cycle analysis for apoptosis in the whole small intestine is shown in Figures 8(a)–8(e). It is evident from Figures 8 and 9 that there were variations in the DNA damage among the concerned developmental stages. Apoptosis was firstly recorded at the developmental stage 55 (12.91 ± 3.99%), followed by highly significant increase at stage 60 (57.26 ± 0.17%). The developmental stage 63 displayed a moderate decrease in terms of apoptosis (45.48 ± 0.81%). At the postmetamorphic stage 66 there was a significant decline in apoptosis (4.43 ± 0.54%) compared to the two metamorphic stages.

## 4. Discussion

The present ultrastructural changes in the developing small intestine of the toad* Bufo regularis* confirm both morphological and histological changes previously reported for the same species [18]. It demonstrates that the toad* Bufo regularis*, as all other vertebrates studied, has two main cell types in the small intestine, that is, columnar and goblet, the former comprising the majority of the epithelial population. The goblet cells are antibacterial and are associated with the aquatic life. The primary epithelium of the tadpole small intestine is similar to its counterpart of other vertebrates.

The evident accumulation of lipid droplets in the small intestine of the postmetamorphic stage 66 confirms the resistance to the possible long periods of starvation. Concomitantly with the start of the larval-to-adult epithelial remodeling in anurans, the fibroblasts actively proliferate and the connective tissue rapidly increases in thickness and cell number [27, 28]. In addition, remarkable ultrastructural changes occur in the epithelial-connective tissue interface and these changes suggest the involvement of the connective tissue in the development of the adult epithelial cell.

Although it remains unknown how apoptosis of the larval epithelium proceeds concomitantly with adult epithelial development, findings of previous electron microscopy studies provide an important clue to its mechanisms. The amphibian intestinal epithelium is separated from the connective tissue by a basal lamina which is usually thin and continuous before and after metamorphosis [27]. When the larval epithelium begins to undergo apoptosis, the basal lamina becomes thick by vigorous folding and remains thick until the larval epithelium disappears [29]. Through the modified basal lamina, subepithelial fibroblasts with well-developed rough endoplasmic reticulum often make cell contacts with the adult epithelial primordia, but not with the degenerating larval epithelium. Consequently, it has been suggested that the thickening of the basal lamina and the cell contacts are related to the primary epithelial cell death and the secondary epithelial cell proliferation, respectively [30]. At later stages, the basal lamina becomes thin beneath the adult epithelium. In addition, the cell contacts and all cell types of the connective tissue, except fibroblasts, decrease in number. By the end of metamorphosis, almost all of the connective tissue cells are ordinary fibroblasts [31]. The basal lamina appears to be much more permeable in spite of its increased thickness during the period of larval epithelial death and adult epithelial proliferation. This permeability is reflected by the frequently observed migration of macrophages across the basal lamina into the degenerating larval epithelium, where they participate in the removal of degenerated cells and the direct contacts between the adult epithelial cells and fibroblast cells on the other side of the basal lamina [27, 32]. Interestingly, the present study of the developing small intestine indicated that both apoptosis of the larval epithelium and active cell proliferation of the adult epithelium coincide with a temporary vigorous folding of the basal lamina underlying the epithelia. It therefore demonstrated a close correlation between the larval epithelial apoptosis and the basal lamina modification.

It has been reported that the larval cell death induced by thyroid hormone can be classified as “apoptosis” [29, 33]. In the intestinal epithelium during amphibian metamorphosis, larval cells undergoing apoptosis coexist with basally disposed undifferentiated cells that will later form the intestinal absorptive epithelium during adulthood (adult cells). The present study has demonstrated that apoptotic cells, with degenerated rough endoplasmic reticulum, swollen/distorted mitochondria, and apoptotic nuclei characterized by irregular nuclear envelop and chromatin condensation, suddenly increase in number only in the larval epithelium at the beginning of the metamorphic climax (at stage 60). In addition, it has revealed that the apoptotic cells are firstly localized in the epithelium of the prometamorphic stage 55 and increased in stages 60 and 63. These results agree well with the observation that the larval epithelial cells are completely replaced by adult cells by stage 66. Not only the present study but also several other previous studies have demonstrated the vital role of apoptosis in the remodeling of certain organs during development [7]. During apoptosis, an endogenous endonuclease is activated that catalyses internucleosomal DNA cleavage to multiple 180–200 bp fragments [34]. Based on that, DNA fragmentation represents molecular evidence for apoptosis [15, 35]. The present results clearly demonstrated this tide correlation where DNA fragmentation was higher in the metamorphic stages (60 and 63) compared to larval stages (50 and 55) and postmetamorphic stage 66.

The apoptotic bodies that resulted from the intestinal remodeling are removed at least in part through phagocytosis by the macrophages which dramatically increase in number concomitantly with larval cell death [3]. While the mechanism which triggers these macrophages to migrate across the basal lamina into the degenerating larval epithelium is unclear, these intraepithelial macrophages become enlarged by engulfing the apoptotic bodies derived from larval epithelial cells and are finally extruded into the intestinal lumen [32]. The present observations indicate that during metamorphosis macrophages-like cells originate and proliferate actively in the connective tissue concomitantly with cell death of the larval epithelium but not among the adult cells. This suggests that macrophages can recognize larval cells before phagocytosing them. In mammals, macrophages are known to possess lectin-like molecules on their cell surface and also recognize cells undergoing apoptosis [4, 8, 36]. Therefore, one can speculate that macrophages recognize modified carbohydrates on the apoptotic cell surface* via *their surface lectin molecules. Similar to the situation with regard to their origin, the ultimate fate of macrophages after phagocytosis of apoptotic bodies is almost unknown. Exceptionally, in the case of the small intestine, they have been observed to become enlarged by engulfed phagosomes and be finally extruded into the gut lumen. As the present results showed, even in the lumen they retain an intact cell membrane and organelles and contain the apoptotic bodies. Earlier studies to explain thyroid hormonal induction of tissue regression during metamorphosis were based on such processes as macrophages infiltration, lysosomal expansion, or activation of lytic enzymes [37]. The evident presence of lysosomes and their association with the metamorphic phase confirm their function in the destruction of cells during normal development. Ultrastructural features observed within the larval epithelium in the present study agree well with that of Wyllie et al. [34] where the apoptotic nuclei displayed condensed chromatin close to nuclear membranes.

Amphibian metamorphosis is a complex process that has been speculated to involve DNA amplification and chromatin rearrangement and therefore the DNA content displayed different values during amphibian development [38]. A few studies have dealt with variation in the amount of DNA during amphibian metamorphosis.* Xenopus laevis *metamorphosis has been reported to be associated with variation in cellular DNA content. Fritz et al. [39] reported that as* X. laevis *tadpoles developed from stage 35 to stage 57, their nuclear fluorescence as measured by flow cytometry increased significantly from stage 46 to 56 and then started to decrease. They suggested that this increase in DNA could be due to gene amplification during larvae development. The highly significant increase in the DNA content of the premetamorphic stage 50 is possibly correlated to the active proliferation at this stage. The major part of the anuran intestinal shortening is caused by DNA fragmentation [6, 40] and the remodeling process depends mainly on apoptosis [7, 28, 41, 42]. Indeed, cellular debris was clearly noticed within the semithin sections of the metamorphic stages of this study. The increased cell proliferation is the major contributor to the temporary thickening of the epithelium at metamorphic climax. In the* Xenopus laevis'* small intestine, apoptotic cells were very few until stage 59 and then rapidly increased in number at stage 60. The larval epithelium cells were totally removed through apoptosis by stage 63, while the adult epithelium cells replacing the larval ones finally differentiated by the end of metamorphosis [6, 7]. In general, apoptosis is one of the most essential phenomena underlying the anuran remodeling from the larval to adult form [3, 28, 43].

In fact, DNA appeared to be more or less intact at both the larval stage 50 and the postmetamorphic stage 66. However, the premetamorphic stage 55 displayed limited degree of DNA fragmentation. In conclusion, the remodeling of the small intestine in anurans in general represents a model for clarifying the mechanism whereby cell death and proliferation are controlled during the remodeling of well-organized tissues. The present work adds new knowledge about both ultrastructure and molecular developmental changes which occur in the intestine of* Bufo regularis* as an example of Anura.
